# Evaluation of a creatinine clearance correction equation based on body fat mass in older Japanese patients with diabetes

**DOI:** 10.3389/fmed.2024.1228383

**Published:** 2024-02-08

**Authors:** Sara Utsumi, Yuki Kondo, Yoshihiko Harada, Akira Yoshida, Hiroyuki Nishimura, Yuki Narita, Tetsumi Irie, Hideaki Jinnouchi, Yoichi Ishitsuka, Sumio Hirata

**Affiliations:** ^1^Department of Clinical Chemistry and Informatics, Graduate School of Pharmaceutical Sciences, Kumamoto University, Kumamoto, Japan; ^2^Diabetes Care Center, Jinnouchi Hospital, Kumamoto, Japan; ^3^Department of Pharmacy, Kumamoto University Hospital, Kumamoto, Japan; ^4^Department of Clinical Pharmaceutical Sciences, Graduate School of Pharmaceutical Sciences, Kumamoto University, Kumamoto, Japan; ^5^Department of Pharmaceutical Packaging Technology, Faculty of Life Sciences, Kumamoto University, Kumamoto, Japan; ^6^Department of Academic Education, I & H Co., Ltd., Ashiya, Japan

**Keywords:** cockcroft-gault equation, correction equation, diabetes, creatinine clearance, fat mass, body composition analyzer

## Abstract

**Background:**

The estimation of creatinine clearance (CCr) in older adult patients with diabetes is subject to deviations from the results of actual measurements because of changes in body composition. In the present study, we aimed to create a correction for the equation used for the estimation of CCr in older adult Asian patients with diabetes using body composition parameters.

**Methods:**

We enrolled 50 older Japanese patients with diabetes in whom the measured values of CCr were compared with values estimated using the Cockcroft-Gault equation. The relationships between the error in the estimated CCr and body composition parameters were investigated, and the Cockcroft-Gault equation was corrected using the appropriate parameters. To evaluate the generalizability of the corrected equation, the utility of the Cockcroft-Gault equation, which was corrected on the basis of body composition measured using a household body composition meter, was also investigated.

**Results:**

Body fat mass (BFM) was closely correlated with the error in the estimated CCr. The BFM-corrected Cockcroft-Gault equation was more accurate than the original equation. Similarly, the error became smaller using BFM measured with a household body composition meter.

**Conclusion:**

The BFM-corrected Cockcroft-Gault equation may provide an accurate method of estimating CCr that can be used in general practice.

## 1 Introduction

Chronic kidney disease is a global public health problem, the global prevalence of which was estimated to be 9.1% in 2017 (1). Aging and diabetes are associated with a deterioration in renal function (2). In developed countries, including Japan, diabetic nephropathy is considered to frequently lead to end-stage renal disease, necessitating dialysis (3). Therefore, accurately estimating renal function is necessary to facilitate the early detection of renal dysfunction and the design of therapeutic approaches that consider the mode of excretion of drugs for patients with diabetes.

In the majority of clinical settings, the Cockcroft-Gault (CG) equation (4), which is designed to estimate creatinine clearance (CCr) using the serum creatinine (SCr) concentration, is often used to evaluate renal function. However, values generated using the CG equation can be affected by patient-specific factors, such as age, body mass, muscle mass, disease, and certain medications (5, 6). Aging and the accompanying changes in body composition affect the SCr concentration because this depends on muscle mass and reduces the accuracy of this method (7, 8). This is a major problem, especially for Asian individuals, who have a lower muscle mass than Westerners (9, 10). In addition, several previous studies have shown that diabetes affects SCr and the accuracy of estimations made using SCr-based equations for Asian people (11, 12). Furthermore, a recent study showed that the relationships between diabetes, muscle loss, and subsequent changes in SCr were affected by aging (13). Therefore, a more accurate method of estimating renal function in older Asian patients with diabetes (e.g., as a simple method of correcting the existing equation) that could be used in clinical practice is required.

In the present study, we compared the accuracies of the original CG equation and a simple corrected version of this equation, based on body composition parameters, in older Japanese adult patients with diabetes. In addition, we investigated the usefulness of the CG equation, which was corrected for body composition parameters measured using a household body composition meter, rather than a precise body composition meter.

## 2 Materials and methods

### 2.1 Study design and participants

We conducted a single-center observational study at Jinnouchi Hospital. The participants were older patients with type 2 diabetes who were admitted to Jinnouchi Hospital between January 2019 and April 2020. Using the definition of the Japanese guidelines for medical treatment and its safety in the elderly (14), older patients in this study were defined as those aged ≥ 65 years. All 50 of the participants provided their written informed consent. The exclusion criteria were as follows: ineligibility for bioelectrical impedance analysis [i.e., patients with a pacemaker, defibrillator, or an artificial joint; acute kidney injury; progressive or terminal cancer; myopathy (e.g., muscular dystrophy); or marked dysuria]; and patients who were undergoing dialysis or taking drugs known to inhibit creatinine secretion in the proximal tubule [e.g., cimetidine (15) and trimethoprim (16)]. The characteristics of the participants are shown in Table 1.

**TABLE 1:** Characteristics of the participants.

	All (*n* = 50)	Men (*n* = 9)	Women (*n* = 21)	*p-*value
Age (years)	72.8 ± 5.9 (65–95)	72.0 ± 4.9 (65–87)	73.9 ± 7.1 (66–95)	0.298
Height (cm)	157.2 ± 8.4 (141.1–175.4)	163.0 ± 5.8 (152.8–175.4)	149.3 ± 3.7 (141.1–155.0)	< 0.001
Body mass (kg)	58.8 ± 8.8 (35.9–79.8)	62.1 ± 8.4 (41.2–79.8)	54.3 ± 7.3 (35.9–69.6)	0.001
Body mass index (kg/m^2^)	23.8 ± 3 (14.6–31.1)	23.4 ± 2.7 (14.6–27.3)	24.4 ± 3.3 (17.1–31.1)	0.273
Obesity, n (%)	22 (44.0)	11 (37.9)	11 (52.4)	0.3912
Duration of diabetes (years)	18.6 ± 11.5 (0–40)	18.4 ± 12.1 (0–37)	19 ± 10.8 (0–40)	0.876
HbA1c (%)	8.8 ± 1.8 (5.8–13.5)	8.5 ± 1.8 (5.8–13.5)	9.2 ± 1.7 (6.5–12.4)	0.159
SCr (mg/dL)	0.9 ± 0.3 (0.6–2.1)	1.1 ± 0.4 (0.7–2.1)	0.8 ± 0.2 (0.6–1.4)	< 0.001
**CKD stage, n (%)**				
G1	0 (0)	0 (0)	0 (0)	
G2	24 (48.0)	13 (44.8)	11 (52.4)	
G3a	20 (40.0)	12 (41.4)	8 (38.1)	0.956
G3b	3 (6.0)	2 (6.9)	1 (4.5)	
G4	3 (6.0)	2 (6.9)	1 (4.5)	
G5	0 (0)	0 (0)	0 (0)	
**Albuminuria category, n (%)**				
A1 (uACR < 30 mg/g, normal to mildly increased)	0 (0)	0 (0)	0 (0)	
A2 (uACR 30–299 mg/g, moderately increased)	24 (48.0)	13 (44.8)	11 (52.4)	0.206
A3 (uACR ≥ 300 mg/g, severely increased)	20 (40.0)	12 (41.4)	8 (38.1)	
BFM (kg)	18.4 ± 6.2 (4.3–31.8)	17.6 ± 5.8 (4.3–28.4)	19.5 ± 6.6 (5.9–31.8)	0.300
Percentage BFM	30.8 ± 8.4 (10.4–49.7)	27.7 ± 7.1 (10.4–39.8)	35.0 ± 8.3 (16.4–49.7)	0.003
FFM (kg)	40.4 ± 6.3 (30–55)	44.5 ± 4.9 (36.9–55.0)	34.8 ± 2.7 (30.0–39.3)	< 0.001
SM (kg)	21.7 ± 3.8 (14.9–30.1)	24.2 ± 2.9 (19.2–30.1)	18.3 ± 1.6 (14.9–20.8)	< 0.001
ASM (kg)	16.2 ± 3.2 (10.4–23.2)	18.4 ± 2.4 (14.7–23.2)	13.3 ± 1.3 (10.4–15.7)	< 0.001
SMI (kg/m^2^)	6.5 ± 0.8 (4.9–8.5)	6.9 ± 0.7 (5.5–8.5)	6.0 ± 0.5 (4.9–6.7)	< 0.001
**Comorbidities, n (%)**				
Hypertension	42 (84.0)	22 (75.9)	20 (95.2)	0.112
Dyslipidemia	39 (78.0)	22 (75.9)	17 (81.0)	0.741
Heart failure	3 (6.0)	1 (3.5)	2 (9.5)	0.565
Diuretics use, n (%)	5 (10.0)	1 (3.5)	4 (19.1)	0.148

Data are expressed as the mean ± SD (minimum–maximum). *p* values are the results of comparisons of men and women using Welch’s *t*-test. SCr, serum creatinine; uACR, urine albumin to creatinine ratio; BFM, fat mass; FFM, fat-free mass; SM, skeletal muscle mass; ASM, appendicular skeletal muscle mass; SMI, skeletal muscle index.

### 2.2 Measurement of actual CCr

The actual creatinine clearance (aCCr) was calculated using the equation shown below.


aCCr⁢(m⁢L/m⁢i⁢n)

$$\;\;=\frac{U\,r\,i\,n\,e\,C\,r\,\left(m\,g/d\,L\right)\times u\,r\,i\,n\,e\,v\,o\,l\,u\,m\,e\,\left(m\,L\right)}{S\,C\,r\,\left(m\,g/d\,L\right)\times d\,u\,r\,a\,t\,i\,o\,n\,o\,f\,u\,r\,i\,n\,e\,c\,o\,l\,l\,e\,c\,t\,i\,o\,n\,\left(m\,i\,n\right)}$$

After emptying the bladder, 24-h urine collection commenced. To measure the duration of urine collection accurately, the times of the commencement and termination of urine collection were recorded for each participant, and the duration of urine collection was calculated in minutes. Therefore, in some of the participants, urine was collected for a slightly longer or shorter time than 1,440 min (24 h), but the aCCr calculated in this way was considered to be more accurate. Consequently, the mean ± SD (minimum-maximum) urine collection time (min) and urine volume (mL) for participants were 1442.8 ± 74.2 (1260–1645) and 1826 ± 707.1 (800–5200), respectively. Furthermore, urinary creatinine excretion (mg/BW/day) was 14.8 ± 4.9 (5.8–34.9). These results indicate that urine collection was nearly complete.

SCr concentrations were measured at the beginning or after the end of urine collection. SCr and urine creatinine concentrations were measured by an enzymatic method using the UniCel DxC600 (Beckman Coulter, Tokyo, Japan).

### 2.3 Estimated CCr

Estimated CCr (eCCr) was calculated using the Cockcroft-Gault equation shown below.


$$\text{eCCr}\left(mL/min\right)=\frac{\left(140-\mathrm{age}\right)\times \mathrm{body}\,\mathrm{mass}\,\left(\text{kg}\right)}{72\times \text{SCr}\,\left(m\,g/d\,L\right)}\left(\times 0.85\mathrm{if}\mathrm{female}\right)$$

### 2.4 Measurement of body composition parameters

Body composition parameters were measured using the InBody^®^ 770 (Inbody Japan Corporation, Tokyo, Japan), which is a high-performance medical body composition meter. We also used the eight-electrode Direct Segmental Multi-frequency bioelectrical impedance analysis method and the RD906 (Tanita Corporation, Tokyo, Japan), which is a household body composition meter. The measurements were made between 16:00 and 17:00 h to avoid the confounding effects of eating and bathing (17). The InBody 770 was used to measure body fat mass (BFM), fat-free mass (FFM), skeletal muscle mass (SM), and appendicular skeletal muscle mass (ASM). FM and SM were also measured using the Tanita RD906.

### 2.5 Statistical analysis

Data are shown as the mean ± standard deviation (SD). To compare the sex differences among the participants, Welch’s *t*-test was used for continuous variables and Fisher’s exact test was used for categorical variables. To evaluate the performance of each equation, Pearson correlation coefficients were calculated. Biases, fixed errors, and proportional errors were calculated using Brand–Altman analysis (18). Applying regression analysis to the Bland-Altman plot, it was determined that proportional bias was present when a significant correlation was observed. The mean absolute error and the percentage of participants with an error within 30% of the aCCr (p30) were used. To compare the equations, paired *t*-tests were performed. The level of significance was set at *p* < 0.05. Statistical analyses were performed using JMP^®^ Pro 16.2 (SAS Institute Inc., Cary, NC, USA).

## 3 Results

### 3.1 Participants’ characteristics

Fifty participants were included in the study, the characteristics of whom are shown in Table 1. Twenty-nine (58%) participants were men. Significant sex differences were identified in height, body mass, body composition (percentage BFM, FFM, SM, ASM, and skeletal muscle index), and SCr concentrations of the participants.

### 3.2 Correlation between aCCr and eCCr

**FIGURE 1:**
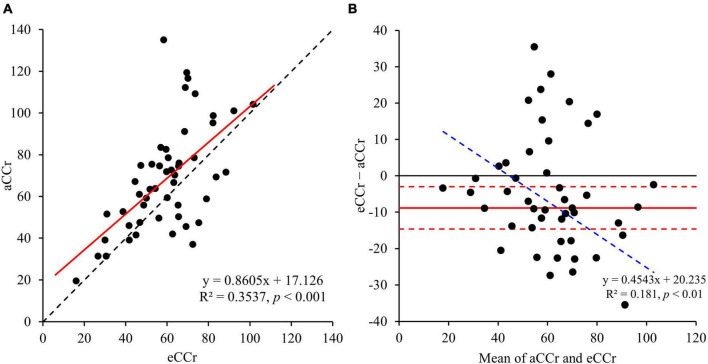
**(A)** Linear regression. y = x is shown by the dotted line and the regression line is shown as the solid red line. **(B)** Bland–Altman plots. The solid red line indicates the mean difference, the red dotted lines represent the upper and lower 95% limits of agreement and the blue dotted line is shown as regression line to indicate proportional error. aCCr, actual creatinine clearance; eCCr, estimated creatinine clearance.

The relationships between aCCr and eCCr are shown in Figure 1. The eCCr was significantly correlated with aCCr (R^2^ = 0.3537, *p* < 0.001, Figure 1A). However, a significant fixed error [bias: mean (95% confidence interval [CI]): –8.82 (–14.6 to –2.99), *p* < 0.004] and a significant proportional error (slope: –0.45, *p* < 0.002) were observed (Figure 1B and Table 2).

**TABLE 2:** Performance of the equations for the estimation of creatinine clearance.

	Bland–Altman analysis			MAE		Within 30% of aCCr (p30)(%)	
	**Bias**	**95% CI**	**slope**		***p* ^a^**		***p* ^a^**
eCCr^a^	−8.82	−14.64 to −2.99	−0.45, *p* < 0.002	16.72	−	68.0	−
eCCr (modified BFM_*medical*_)	−3.64	−8.59 to 1.31	−0.40, *p* < 0.001	12.61	0.005	78.0	0.132
eCCr (modified BFM_*household*_)	−3.87	−8.85 to 1.11	−0.43, *p* < 0.001	13.33	0.017	78.0	0.132

*^a^p*-values are shown for the MAE and within 30% of aCCr with respect to eCCr versus modified eCCr. MAE, mean absolute error; 95% CI, 95% confidence interval; aCCr, actual creatinine clearance; eCCr, estimated creatinine clearance; BFM_*medical*_, fat mass measured using InBody; BFM_*household*_, fat mass measured using a household body composition analyzer.

### 3.3 Relationships of the difference between eCCr and aCCr with body composition parameters

**FIGURE 2:**
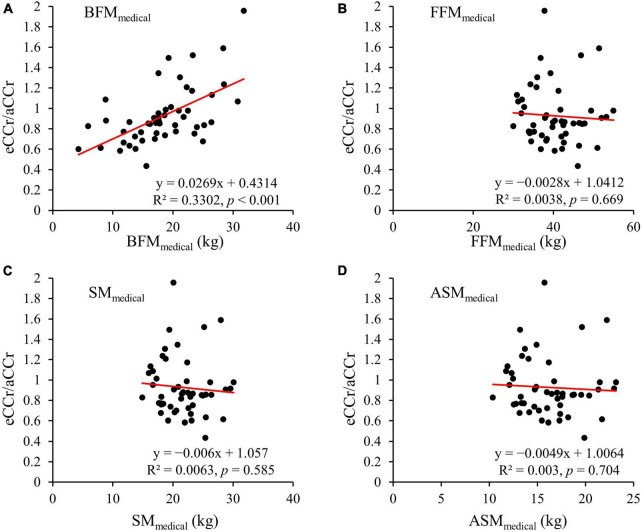
**(A)** BFM_*Medical*_. **(B)** FF_*Medical*_. **(C)** SM_*Medical*_. **(D)** ASM_*Medical*_. y = x = is shown as the dotted line and the regression line is shown as the solid red line. aCCr, actual creatinine clearance; eCCr, estimated creatinine clearance; BFM_*Medical*_, fat mass measured using InBody; FF_*Medical*_, fat-free mass measured using InBody; SM_*Medical*_, skeletal mass measured using InBody; ASM_*Medical*_, appendicular skeletal mass measured using InBody.

The relationships of eCCr/aCCr with the body composition parameters measured using the InBody are shown in Figure 2. BFM_*medical*_ was significantly correlated with the difference between the measured and estimated values (R^2^ = 0.3302, *p* < 0.001), and the equation generated is shown below. None of the other body composition parameters were correlated with this difference.


eCCraCCr=0.0269276×BFMmedical⁢ 0.4313819⁢[E⁢q⁢u⁢a⁢t⁢i⁢o⁢n⁢1]

### 3.4 Relationships between aCCr and eCCr modified using BFM_*medical*_

To obtain a more accurate eCCr equation, Equation 1 was transformed to obtain the following equation.


$$\text{eCCr}\,\left(\mathrm{modified}\,{\mathrm{BFM}}_{\text{medical}}\right)=\,\frac{\text{eCCr}}{0.0269276\times {\text{BFM}}_{\text{medical}}}+0.4313819\,\left[E\,q\,2\right]$$

**FIGURE 3:**
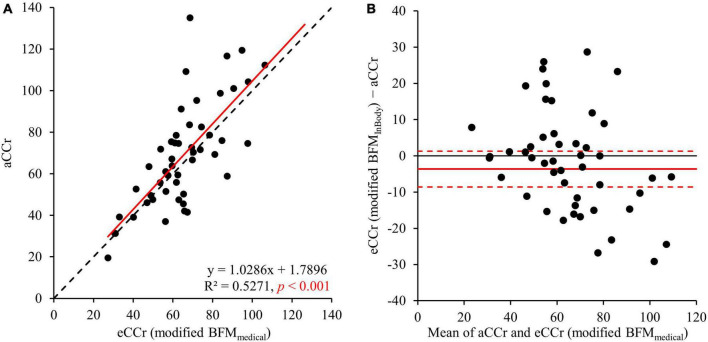
Relationships between aCCr and eCCr modified using BFM_*Medical*_. **(A)** Linear regression. y = x is shown as the dotted line and the regression line is shown as the solid red line. **(B)** Bland–Altman plots. The solid red line indicates the mean difference and the dotted lines represent the upper and lower 95% limits of agreement. aCCr, actual creatinine clearance; eCCr, estimated creatinine clearance; BFM_*Medical*_, fat mass measured using InBody.

The correlation between aCCr and eCCr (modified BFM_*medical*_) was significant (R^2^ = 0.5271, *p* < 0.001, Figure 3). In addition, significant fixed errors that were present in the original equation were not present with eCCr (modified BFM_*medical*_) (bias: mean [95% CI]: –3.64 [–8.59 to 1.31], *p* < 0.004; Figure 3B and Table 2). Furthermore, the mean absolute error of eCCr (modified BFM_*medical*_) was significantly lower than that of eCCr (Table 2). There was also a tendency toward greater accuracy as determined using the values obtained within 30% of the aCCR for eCCr (modified BFM_*medical*_) and eCCr (p30: 78.0%, *p* = 0.132; Table 2).

### 3.5 Comparison of eCCr (modified BFM_*medical*_) and eCCr (modified BFM_*household*_)

**FIGURE 4:**
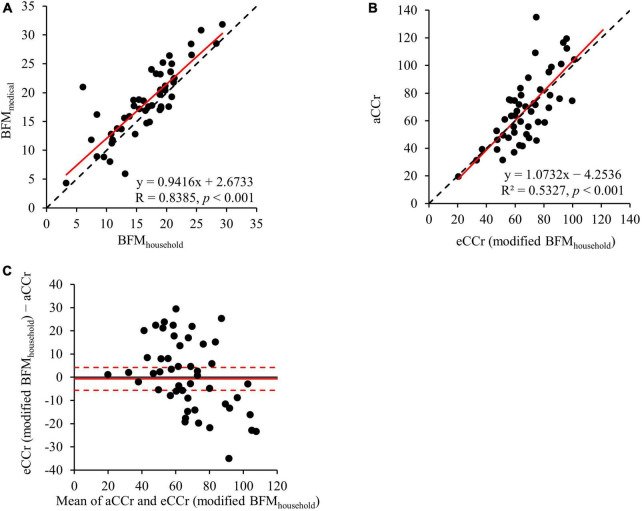
Comparison of eCCr (modified BFM_*Medical*_) and eCCr (modified BFM_*household*_). **(A)** Linear regression of the relationship between BFM_*Medical*_ and BFM_*household*_. y = x is shown as the dotted line and the regression line is shown as the solid red line. **(B)** Linear regression of the relationship between aCCr and eCCr (modified BFM_*household*_). y = x is shown as the dotted line and the regression line is shown as the solid red line. **(C)** Bland–Altman plots for aCCr and eCCr (modified BFM_*household*_). The solid red line indicates the mean difference and the dotted lines represent the upper and lower 95% limits of agreement. aCCr, actual creatinine clearance; eCCr, estimated creatinine clearance; BFM_*Medical*_, fat mass measured using InBody; BFM_*household*_, fat mass measured using a household body composition analyzer.

The results of the comparison of eCCr (modified BFM_*medical*_) and eCCr (modified BFM_*household*_) are shown in Figure 4. BFM_*household*_ was significantly correlated with BFM_*medical*_ (R = 0.8385, *p* < 0.001) (Figure 4A). The eCCr (modified BFM_*household*_) was calculated by substituting BFM_*household*_ into Equation 2. Therefore, eCCr (modified BFM_*household*_) was also significantly correlated with aCCr (R^2^ = 0.5327, *p* < 0.001) (Figure 4B). In addition, eCCr (modified BFM_*household*_) performed similarly to eCCr (modified BFM_*medical*_) (Figure 4C and Table 2).

## 4 Discussion

This study showed that the original Cockcroft-Gault equation underestimated CCr in older Japanese adult patients with diabetes and that BFM was closely correlated with the difference between aCCr and eCCr. In addition, the use of modified equations using BFM measured using medical devices reduced this error. Finally, we showed that this error was also reduced by the use of a formula adjusted using BFM measured with a household body composition meter.

Obesity is one of the causes of the underestimation of renal dysfunction when using the CG equation (19), and the prevalence of obesity in patients with diabetes is high in Japan (20). Similarly, the prevalence of obesity (44%) in the participants in the present study was high. Nevertheless, the eCCr for older adult patients with diabetes was underestimated using the CG equation (Figure 1 and Table 2). Several previous studies (21–23) have shown that SCr-based formulae underestimate the renal function of Asian people, including Japanese people. In particular, the CG equation has been shown to underestimate CCr in older Japanese adult patients (24). Therefore, eCCr is not always overestimated in older Asian patients with diabetes.

To improve the accuracy of the estimation of CCr using the CG equation, we investigated the relationships between body composition parameters measured using a medical device and the error in the eCCr (eCCr/aCCr). We found that BFM was closely correlated with the error in eCCr and that eCCr was underestimated in participants with a low BFM and overestimated in those with a high BFM (Figure 2A). Interestingly, the indices of muscle mass (FFM, SM, and ASM), which should closely correlate with creatinine production, did not correlate with the error in eCCr (Figure 2D). Otani et al. (8) previously reported the usefulness of correcting eCCr using BFM for bedridden older Japanese people, and suggested that the high relative adiposity of bedridden patients may explain the error in eCCr. In general, Japanese patients with diabetes have a high percentage of body fat (25) and a low percentage of muscle mass (26). Furthermore, aging is associated with an increase in the percentage of body fat (27). This result has also been demonstrated in studies of Japanese (28) and Singaporean (29) adult cohorts. This evidence suggests that older Asians have a high body fat percentage (i.e., a relatively high amount of body fat per unit body weight). Consistent with this evidence, the participants in the present study had a high percentage of body fat (Table 1). These findings may explain why BFM correlates with the error in eCCr in older patients with diabetes. Therefore, we modified the CG equation using BFM, which yielded a superior predictive performance to the original CG equation (Figure 2 and Table 2). In addition, BFM contributed more strongly to the errors of the estimation equation than any other body composition parameter (Supplementary Table 1), and no significant partial correlations were observed with the other parameters (Supplementary Tables 3, 4). These results suggest that the correction of the CG equation with BFM improves the assessment of renal function in older adult Japanese patients with diabetes.

Medical body composition meters, such as the InBody770, can be used to accurately measure body composition parameters with the eight-electrode Direct Segmental Multi-frequency bioelectrical impedance analysis method (30) and dual-energy X-ray absorptiometry (19). Therefore, these meters have been used in many studies, including in the field of nephrology (7, 31–33). However, such meters are expensive and are thus only available in some medical institutions. In contrast, household body composition meters, such as the Tanita RD906, are cheaper than medical body composition analyzers and are easy to use, such that they can be used in a wider range of facilities. Therefore, to render the BFM-corrected CG equation more widely applicable, we next investigated whether BFM measured using a household body composition meter provides a useable alternative to BFM measured using a medical device for the modification of the CG equation. We found that the BFM_*household*_ values were similar to the BFM_*medical*_ values (Figure 4A). In addition, the CG equation corrected using BFM_*household*_ was similarly accurate to the equation in which BFM_*medical*_ was used (Figures 4B, C, and Table 2). Moreover, the improvement in accuracy of the BFM-corrected CG equation was consistent, regardless of SCr concentrations and gender and this trend was more pronounced in men and patients with chronic kidney disease (Supplementary Table 2). This gender difference may be explained by the gender disparity in the prevalence of sarcopenia among older adults. A previous report (34) indicated a higher prevalence of sarcopenia in older Japanese men than in women. Therefore, the larger difference between eCCr and aCCr in men might have led to a more pronounced correction in the estimation equations. These results suggest that the use of the CG equation corrected for BFM is suitable for use not only in specialist medical institutions, but also in wider clinical settings, such as in community pharmacies, for patients with chronic kidney disease.

The present study has some limitations. First, the participants were exclusively Japanese. Therefore, whether the present findings can be generalized to individuals of other ethnicities, including members of other Asian populations, is unclear. Second, the effects of circadian variation and the season on BFM were not investigated. Measurements of body composition were performed between 16:00 and 17:00 h to minimize the effects of bathing and eating, but whether our findings are applicable in other situations is unclear. Third, we aimed to correct estimations of CCr; therefore, this method is unlikely to be applied to the estimation of the glomerular filtration rate. The expression of organic cation transporters, which are one type of creatinine transporter (35), has been reported to be high in the presence of oxidative stress and high concentrations of advanced glycation end-products in rats (36). On the basis of these findings, Tsuda et al. suggested that patients with diabetes have greater tubular secretion of creatinine (11). Therefore, the equation corrected for BFM may only be applicable to eCCr. Fourth, there was a proportional error associated with the corrected equation. The correction for BFM improved the fixed error associated with the CG equation, but did not affect the proportional error (Table 2). Fifth, the presence of albuminuria reduced the improvement of accuracy in the BFM_*household*_-corrected CG equation (Supplementary Table 2). The definitive mechanism is unknown, but using the correction equation is an important limitation. Sixth, although we examined confounders, we cannot rule out the existence of unknown confounders owing to the study design in this study. Indeed, the potential association between nutritional parameters and errors in renal function estimation equations cannot be denied. The nutritional parameters measured in this study, such as total cholesterol, serum albumin, serum iron, and various electrolyte concentrations, did not show a significant association with equation errors (Supplementary Tables 3, 4). However, other nutritional parameters, including transthyretin, total lymphocyte count, and dietary intake, have not been investigated. Seventh, although BFM_*household*_ and BFM_*medical*_ were closely correlated, the differences in the values obtained were large in some of the participants. We attempted to determine the causes of these differences, but could not identify a clear explanation. Eighth, the investigation of factors that could influence body composition is insufficient. In this study, there were few participants with a history of heart failure or the use of diuretics, factors that could potentially affect body composition. Therefore, it is unclear whether the results of this study are consistent in patients with these conditions. Finally, this study is a single-center study. The results of this study need to be validated in future additional studies, involving multiple facilities, to avoid excessive generalization of the current findings. To resolve these limitations, further studies are required.

## 5 Conclusion

This study shows that fat mass can be used to improve the accuracy with which CCr is estimated in older Japanese adult patients with diabetes. This result helps improve the underestimated renal function in individuals with diabetes, which is a prevailing foundational condition of renal impairment. Therefore, the improvement has the potential to enhance the refinement of drug dosages contingent upon CCr. In addition, the accuracy can be improved even when fat mass is assessed using a low-cost household body composition analyzer. The simplification of body composition assessment in this study is promising for clinical implementation and research of estimating body composition-based renal function in the field of nephrology. Although further studies to validate the results of this study are warranted, we consider that this method of modifying the CG equation based on BFM should be clinically useful.

## Data availability statement

The raw data supporting the conclusions of this article will be made available by the authors, without undue reservation.

## Ethics statement

The studies involving humans were approved by the Independent Ethics Committee of Kumamoto University Faculty of Life Sciences and Jinnouchi Hospital Institutional Review Board. The studies were conducted in accordance with the local legislation and institutional requirements. The participants provided their written informed consent to participate in this study.

## Author contributions

SH conceived and designed the study. YK, TI, YI, and SH were responsible for project administration, responsible for supervision, and reviewed and edited the manuscript. SU, YH, AY, HN, and HJ were responsible for collecting data. SU, YH, and YK were responsible for the formal analysis. SU and YK were responsible for writing the original draft of the manuscript and the data curation. All authors were responsible for the conceptualization of the study and the methodology and approved the submitted version
