# Supplementary material for: Evaluation of a creatinine clearance correction equation based on body fat mass in older Japanese patients with diabetes
Source: Front Med (Lausanne). 2024 Feb 8;11:1228383. doi: 10.3389/fmed.2024.1228383 (PMC10881716; doi:10.3389/fmed.2024.1228383)
Supplement: Supplementary file 1 [file Data_Sheet_1.pdf]

Supplemental Table 1 Variables selection using random forests for CG equation correction

Rank	Variable	Contribution	Portion
1	BFM_{InBody}	0.993395	0.6574
2	SM_{InBody}	0.171507	0.1135
3	SMI_{InBody}	0.158042	0.1046
4	FFM_{InBody}	0.096525	0.0639
5	ASM_{InBody}	0.09159	0.0606

BFM=fat mass. SM=skeletal muscle mass. SMI=skeletal muscle index. FFM=fat-free mass. ASM=appendicular skeletal muscle mass.

Supplemental Table 2 Performance of the equation for estimating creatinine clearance by serum creatinine level, CKD, albuminuria, gender

		Bland-Altman analysis			MAE		Within 30% of aCCr （p30） (%)	
		Bias	95% CI	slope		p [a]		p [a]
Low-level sCr ($sCr \le 0.93$) n = 30	eCCr[a]	−7.47	−15.3 to 0.44	−0.63, $p < 0.005$	17.97	-	63.3	-
	eCCr (modified $BFM_{medical}$)	−1.44	−8.01 to 5.12	−0.51, $p < 0.003$	13.86	0.065	73.3	0.257
	eCCr (modified $BFM_{household}$)	0.00	−6.45 to 6.45	−0.51, $p < 0.002$	14.58	0.091	73.3	0.257
High-level sCr ($sCr > 0.93$) n = 20	eCCr[a]	−10.84	−20.10 to −1.59	−0.61, $p < 0.013$	14.84	-	75.0	-
	eCCr (modified $BFM_{medical}$)	−6.94	−14.93 to 3.82	−0.69, $p < 0.001$	10.74	0.014	85.0	0.317
	eCCr (modified $BFM_{household}$)	−1.74	−10.11 to 6.62	−0.60, $p < 0.005$	11.45	0.089	85.0	0.317
Non-CKD ($eGFR \ge 60$) n = 24	eCCr[a]	−9.54	−18.16 to −0.94	−0.78, $p < 0.004$	17.45	-	70.8	-
	eCCr (modified $BFM_{medical}$)	−2.90	−10.76 to 4.96	−0.68, $p < 0.004$	14.73	0.301	75.0	0.654
	eCCr (modified $BFM_{household}$)	−0.55	−7.94 to 6.84	−0.73, $p < 0.001$	14.36	0.205	75.0	0.654
CKD ($eGFR < 60$) n = 26	eCCr[a]	−8.15	−16.62 to 0.32	−0.63, $p < 0.016$	16..05	-	65.4	
	eCCr (modified $BFM_{medical}$)	−4.33	−11.02 to 2.36	−0.71, $p < 0.001$	10.65	< 0.001	80.8	0.103
	eCCr (modified $BFM_{household}$)	−0.83	−7.92 to 6.26	−0.70, $p < 0.001$	12.41	0.023	80.8	0.103

Non-Albuminuria (uACR < 30 mg/g) n = 29	eCCr[a]	−12.01	−19.02 to −5.12	−0.44, $p < 0.015$	17.64	-	58.6	-
	eCCr (modified $BFM_{medical}$)	−4.01	−10.43 to 2.24	−0.40, $p < 0.01$	13.07	0.042	79.31	0.058
	eCCr (modified $BFM_{household}$)	−2.58	−8.41 to 3.24	−0.39, $p < 0.007$	12.77	0.011	82.8	0.020
Albuminuria ((uACR ≥ 30 mg/g) n = 21	eCCr[a]	−4.33	−14.77 to 6.10	−0.45, $p = 0.080$	15.45	-	81.0	-
	eCCr (modified $BFM_{medical}$)	−3.14	−11.83 to 5.55	−0.11, $p < 0.050$	11.98	0.046	76.2	0.317
	eCCr (modified $BFM_{household}$)	1.91	−7.16 to 10.98	−0.49, $p < 0.050$	14.01	0.531	71.4	0.157
Men n = 29	eCCr[a]	−13.91	−21.87 to −5.97	−0.51, $p < 0.007$	18.14	-	69.0	-
	eCCr (modified $BFM_{medical}$)	−6.77	−13.32 to −0.21	−0.35, $p < 0.015$	12.42	0.003	86.2	0.059
	eCCr (modified $BFM_{household}$)	−1.71	−8.67 to 5.26	−0.47, $p < 0.004$	13.54	0.020	82.8	0.157
Women n = 21	eCCr[a]	−1.78	−10.02 to 6.47	−0.26, $p = 0.275$	14.77	-	66.7	
	eCCr (modified $BFM_{medical}$)	0.67	−7.12 to 8.46	−0.43, $p = 0.056$	12.87	0.165	66.7	1.000
	eCCr (modified $BFM_{household}$)	0.70	−6.71 to 8.10	−0.43, $p < 0.05$	13.05	0.127	71.4	0.564

Based on the reference mean serum creatinine level (0.93), the participants were divided into two groups.
[a] *P*-values are shown for MAE and within 30% of aCCr with respect to eCCr *versus* modified eCCr. 95% CI, 95% confidence interval; aCCr, actual creatinine clearance; eCCr, estimated creatinine clearance; BFM_{InBody}, fat mass measured using InBody; $BFM_{household}$, fat mass measured using a household body composition analyzer.
