# Supplementary material for: Evaluation of a creatinine clearance correction equation based on body fat mass in older Japanese patients with diabetes
Source: Front Med (Lausanne). 2024 Feb 8;11:1228383. doi: 10.3389/fmed.2024.1228383 (PMC10881716; doi:10.3389/fmed.2024.1228383)
Supplement: Supplementary file 2 [file Data_Sheet_2.docx]

| **Supplemental Table 3 partial correlation coefficient** | | | | | | | | | | | |  |  |  |  |  |  |  |  |  |  |  |  |  |
| --- | --- | --- | --- | --- | --- | --- | --- | --- | --- | --- | --- | --- | --- | --- | --- | --- | --- | --- | --- | --- | --- | --- | --- | --- |
|  | Age | Tall | HbA1c (NGSP) | eCCr/aCCr | FM_InBody_ | ASM_InBody_ | FFM_InBody_ | SM_InBody_ | Serum albumin | AST | ALT | γ-GTP | ALP | LDH | Total choresterol | HDL | LDL | Triglyceride | Ureiic acid | Na | Cl | K | Ca | serum iron |
| Age |  | 0.068134 | 0.258657911 | 0.05479661 | -0.375811829 | 0.07593 | 0.35218 | -0.41753 | 0.036432009 | 0.1277 | -0.22983 | 0.048996 | -0.26806 | 0.052125 | 0.140569509 | -0.14419 | -0.18729 | -0.0723939 | 0.0029959 | 0.117239 | -0.00769 | 0.167246 | -0.29005 | 0.062654967 |
| Tall | 0.068134 |  | -0.088000678 | 0.01920499 | -0.069962294 | 0.372776 | -0.08924 | 0.048709 | 0.014375814 | -0.055 | 0.128099 | 0.008101 | -0.03839 | -0.28451 | 0.129595723 | -0.08518 | -0.10895 | -0.0826823 | 0.356499 | 0.207646 | -0.4119 | -0.14081 | -0.07522 | -0.090198463 |
| HbA1c (NGSP) | 0.258658 | -0.088 |  | -0.3130594 | 0.331004571 | 0.070973 | -0.1276 | 0.103913 | -0.183198316 | -0.347 | 0.414962 | 0.000612 | 0.140108 | -0.13834 | -0.108073341 | 0.184738 | 0.221013 | 0.06789084 | 0.0174736 | 0.252987 | -0.32287 | -0.14873 | 0.294883 | -0.075619975 |
| eCCr/aCCr | 0.054797 | 0.019205 | -0.313059387 |  | 0.573429683 | -0.18327 | 0.149147 | -0.10362 | -0.254976479 | -0.242 | 0.190395 | 0.01794 | -0.11685 | 0.187296 | 0.104056586 | -0.00167 | -0.05377 | 0.11837837 | -0.223923 | 0.31825 | -0.26744 | 0.020602 | 0.287597 | -0.332893096 |
| FM_InBody_ | -0.37581 | -0.06996 | 0.331004571 | 0.57342968 |  | 0.327685 | -0.01921 | -0.08197 | 0.208602393 | 0.2095 | -0.23465 | 0.062455 | -0.00669 | 0.084431 | 0.076619688 | -0.2672 | -0.1362 | 0.0228723 | 0.2392096 | -0.12012 | 0.177018 | 0.027166 | -0.33256 | 0.159418443 |
| ASM_InBody_ | 0.07593 | 0.372776 | 0.070972678 | -0.1832702 | 0.327684786 |  | 0.339973 | -0.05166 | -0.10087833 | -0.051 | 0.114539 | -0.06566 | 0.02096 | 0.102458 | -0.079507734 | 0.082576 | 0.041999 | 0.16030405 | -0.081316 | -0.04027 | -0.08292 | -0.01141 | 0.01559 | -0.070546366 |
| FFM_InBody_ | 0.35218 | -0.08924 | -0.127601614 | 0.14914684 | -0.019212831 | 0.339973 |  | 0.952544 | 0.035618238 | 0.1294 | -0.2672 | -0.08592 | 0.064558 | 0.258171 | -0.323907336 | 0.245721 | 0.313787 | 0.03456994 | -0.080916 | -0.18092 | 0.04909 | -0.53928 | 0.228737 | -0.273332564 |
| SM_InBody_ | -0.41753 | 0.048709 | 0.103912944 | -0.1036224 | -0.081970106 | -0.05166 | 0.952544 |  | -0.014117813 | -0.133 | 0.243186 | 0.133414 | -0.09462 | -0.28423 | 0.356060816 | -0.27592 | -0.33771 | -0.0854681 | 0.0738903 | 0.191274 | 0.001144 | 0.586172 | -0.22917 | 0.318371998 |
| Serum albumin | 0.036432 | 0.014376 | -0.183198316 | -0.2549765 | 0.208602393 | -0.10088 | 0.035618 | -0.01412 |  | -0.282 | 0.205043 | 0.290313 | -0.27665 | 0.16779 | -0.052854201 | 0.171568 | 0.04614 | 0.21268542 | -0.145509 | 0.377094 | -0.30358 | 0.177882 | 0.673996 | 0.143443105 |
| AST | 0.127674 | -0.05536 | -0.346733155 | -0.2422273 | 0.209546377 | -0.05125 | 0.129445 | -0.13344 | -0.281849661 |  | 0.647952 | 0.670428 | -0.05363 | 0.234481 | -0.107445797 | 0.29206 | 0.071543 | 0.33222871 | -0.077261 | 0.297417 | -0.25732 | 0.272243 | 0.296355 | -0.021344658 |
| ALT | -0.22983 | 0.128099 | 0.414962256 | 0.19039511 | -0.234654257 | 0.114539 | -0.2672 | 0.243186 | 0.205043481 | 0.648 |  | -0.15473 | -0.06997 | 0.115069 | 0.023430054 | -0.12448 | -0.09029 | -0.149268 | -0.188295 | -0.23328 | 0.23596 | -0.16353 | -0.19802 | 0.015176159 |
| γ-GTP | 0.048996 | 0.008101 | 0.00061178 | 0.01793951 | 0.062454614 | -0.06566 | -0.08592 | 0.133414 | 0.290312618 | 0.6704 | -0.15473 |  | 0.36813 | -0.35946 | 0.047209114 | -0.16087 | 0.077583 | -0.2207649 | 0.3098725 | -0.04603 | 0.039187 | -0.33831 | -0.27467 | -0.033391898 |
| ALP | -0.26806 | -0.03839 | 0.140107705 | -0.1168489 | -0.006693023 | 0.02096 | 0.064558 | -0.09462 | -0.276649229 | -0.054 | -0.06997 | 0.36813 |  | 0.152087 | -0.011939861 | 0.026324 | -0.00486 | -0.0079149 | -0.423342 | 0.118112 | -0.10811 | 0.058623 | 0.213022 | -0.123573631 |
| LDH | 0.052125 | -0.28451 | -0.138340325 | 0.18729551 | 0.084431437 | 0.102458 | 0.258171 | -0.28423 | 0.167790319 | 0.2345 | 0.115069 | -0.35946 | 0.152087 |  | 0.212540214 | -0.18019 | -0.0951 | -0.3792513 | 0.3725161 | 0.162946 | -0.14942 | 0.041429 | -0.28412 | 0.282817161 |
| Total choresterol | 0.14057 | 0.129596 | -0.108073341 | 0.10405659 | 0.076619688 | -0.07951 | -0.32391 | 0.356061 | -0.052854201 | -0.107 | 0.02343 | 0.047209 | -0.01194 | 0.21254 |  | 0.900537 | 0.955205 | 0.64635633 | -0.13462 | -0.13542 | 0.146834 | -0.13771 | 0.211178 | -0.007800062 |
| HDL | -0.14419 | -0.08518 | 0.184738295 | -0.0016738 | -0.267201725 | 0.082576 | 0.245721 | -0.27592 | 0.17156774 | 0.2921 | -0.12448 | -0.16087 | 0.026324 | -0.18019 | 0.900537123 |  | -0.85487 | -0.6566957 | 0.1847734 | 0.019004 | -0.08081 | 0.043201 | -0.24732 | 0.015352343 |
| LDL | -0.18729 | -0.10895 | 0.221012811 | -0.0537693 | -0.136198009 | 0.041999 | 0.313787 | -0.33771 | 0.046140239 | 0.0715 | -0.09029 | 0.077583 | -0.00486 | -0.0951 | 0.955204905 | -0.85487 |  | -0.5568908 | 0.0495363 | 0.026251 | -0.10166 | 0.136884 | -0.1884 | -0.009122201 |
| Triglyceride | -0.07239 | -0.08268 | 0.067890844 | 0.11837837 | 0.022872298 | 0.160304 | 0.03457 | -0.08547 | 0.212685415 | 0.3322 | -0.14927 | -0.22076 | -0.00791 | -0.37925 | 0.646356331 | -0.6567 | -0.55689 |  | 0.0476625 | -0.14297 | 0.137386 | -0.06323 | -0.211 | 0.129869171 |
| Ureiic acid | 0.002996 | 0.356499 | 0.017473634 | -0.2239229 | 0.239209607 | -0.08132 | -0.08092 | 0.07389 | -0.145508965 | -0.077 | -0.18829 | 0.309872 | -0.42334 | 0.372516 | -0.134620495 | 0.184773 | 0.049536 | 0.04766253 |  | -0.21512 | 0.306396 | 0.095954 | 0.315552 | -0.298560687 |
| Na | 0.117239 | 0.207646 | 0.252986895 | 0.31824954 | -0.120124693 | -0.04027 | -0.18092 | 0.191274 | 0.377094336 | 0.2974 | -0.23328 | -0.04603 | 0.118112 | 0.162946 | -0.13541767 | 0.019004 | 0.026251 | -0.1429698 | -0.215123 |  | 0.676793 | -0.20653 | 0.007869 | 0.003090568 |
| Cl | -0.00769 | -0.4119 | -0.322865176 | -0.2674433 | 0.177018477 | -0.08292 | 0.04909 | 0.001144 | -0.303581329 | -0.257 | 0.23596 | 0.039187 | -0.10811 | -0.14942 | 0.146834051 | -0.08081 | -0.10166 | 0.13738608 | 0.3063961 | 0.676793 |  | -0.08597 | 0.091378 | -0.153332582 |
| K | 0.167246 | -0.14081 | -0.14873257 | 0.02060167 | 0.027165687 | -0.01141 | -0.53928 | 0.586172 | 0.177882224 | 0.2722 | -0.16353 | -0.33831 | 0.058623 | 0.041429 | -0.137713924 | 0.043201 | 0.136884 | -0.0632262 | 0.0959544 | -0.20653 | -0.08597 |  | 0.116986 | -0.295686091 |
| Ca | -0.29005 | -0.07522 | 0.294882982 | 0.28759675 | -0.33256324 | 0.01559 | 0.228737 | -0.22917 | 0.673996243 | 0.2964 | -0.19802 | -0.27467 | 0.213022 | -0.28412 | 0.211177871 | -0.24732 | -0.1884 | -0.2110009 | 0.3155523 | 0.007869 | 0.091378 | 0.116986 |  | 0.069920081 |
| serum iron | 0.062655 | -0.0902 | -0.075619975 | -0.3328931 | 0.159418443 | -0.07055 | -0.27333 | 0.318372 | 0.143443105 | -0.021 | 0.015176 | -0.03339 | -0.12357 | 0.282817 | -0.007800062 | 0.015352 | -0.00912 | 0.12986917 | -0.298561 | 0.003091 | -0.15333 | -0.29569 | 0.06992 |  |
| Text color red: Significant difference (*p* < 0.05) | | | | | | | | | | | | | | | | | | | | | | | | |
