# Supplementary material for: Evaluation of a creatinine clearance correction equation based on body fat mass in older Japanese patients with diabetes
Source: Front Med (Lausanne). 2024 Feb 8;11:1228383. doi: 10.3389/fmed.2024.1228383 (PMC10881716; doi:10.3389/fmed.2024.1228383)
Supplement: Supplementary file 3 [file Data_Sheet_3.docx]

| **Supplemental Table 4 *p*-value of partial correlation** | | | | | | | | | | | |  |  |  |  |  |  |  |  |  |  |  |  |  |
| --- | --- | --- | --- | --- | --- | --- | --- | --- | --- | --- | --- | --- | --- | --- | --- | --- | --- | --- | --- | --- | --- | --- | --- | --- |
|  | Age | Tall | HbA1c (NGSP) | eCCr/aCCr | FM_InBody_ | ASM_InBody_ | FFM_InBody_ | SM_InBody_ | Serum albumin | AST | ALT | γ-GTP | ALP | LDH | Total choresterol | HDL | LDL | Triglyceride | Ureiic acid | Na | Cl | K | Ca | serum iron |
| Age | . | 0.7305 | 0.1838 | 0.7818 | 0.0487 | 0.701 | 0.0661 | 0.0271 | 0.854 | 0.5173 | 0.2394 | 0.8045 | 0.1678 | 0.7922 | 0.4756 | 0.4641 | 0.3399 | 0.7143 | 0.9879 | 0.5524 | 0.969 | 0.395 | 0.1343 | 0.7514 |
| Tall | 0.7305 | . | 0.6561 | 0.9227 | 0.7235 | 0.0507 | 0.6515 | 0.8056 | 0.9421 | 0.7796 | 0.5159 | 0.9674 | 0.8462 | 0.1423 | 0.511 | 0.6665 | 0.581 | 0.6757 | 0.0626 | 0.289 | 0.0294 | 0.4748 | 0.7036 | 0.6481 |
| HbA1c (NGSP) | 0.1838 | 0.6561 | . | 0.1048 | 0.0853 | 0.7197 | 0.5176 | 0.5987 | 0.3508 | 0.0707 | 0.0281 | 0.9975 | 0.477 | 0.4827 | 0.5841 | 0.3466 | 0.2584 | 0.7314 | 0.9297 | 0.194 | 0.0938 | 0.45 | 0.1277 | 0.7021 |
| eCCr/aCCr | 0.7818 | 0.9227 | 0.1048 | . | 0.0014 | 0.3506 | 0.4488 | 0.5998 | 0.1904 | 0.2143 | 0.3318 | 0.9278 | 0.5537 | 0.3399 | 0.5982 | 0.9933 | 0.7858 | 0.5485 | 0.252 | 0.0988 | 0.1689 | 0.9171 | 0.1378 | 0.0835 |
| FM_InBody_ | 0.0487 | 0.7235 | 0.0853 | 0.0014 | . | 0.0887 | 0.9227 | 0.6784 | 0.2868 | 0.2845 | 0.2294 | 0.7522 | 0.973 | 0.6693 | 0.6984 | 0.1693 | 0.4895 | 0.908 | 0.2202 | 0.5426 | 0.3675 | 0.8909 | 0.0838 | 0.4178 |
| ASM_InBody_ | 0.701 | 0.0507 | 0.7197 | 0.3506 | 0.0887 | . | 0.0767 | 0.794 | 0.6095 | 0.7957 | 0.5617 | 0.7399 | 0.9157 | 0.6039 | 0.6876 | 0.6761 | 0.832 | 0.4151 | 0.6808 | 0.8388 | 0.6749 | 0.954 | 0.9372 | 0.7213 |
| FFM_InBody_ | 0.0661 | 0.6515 | 0.5176 | 0.4488 | 0.9227 | 0.0767 | . | <.0001 | 0.8572 | 0.5115 | 0.1693 | 0.6638 | 0.7441 | 0.1847 | 0.0927 | 0.2075 | 0.1039 | 0.8614 | 0.6823 | 0.3569 | 0.8041 | 0.0031 | 0.2417 | 0.1593 |
| SM_InBody_ | 0.0271 | 0.8056 | 0.5987 | 0.5998 | 0.6784 | 0.794 | <.0001 | . | 0.9432 | 0.4985 | 0.2124 | 0.4985 | 0.632 | 0.1427 | 0.0629 | 0.1553 | 0.0788 | 0.6654 | 0.7086 | 0.3295 | 0.9954 | 0.001 | 0.2408 | 0.0987 |
| Serum albumin | 0.854 | 0.9421 | 0.3508 | 0.1904 | 0.2868 | 0.6095 | 0.8572 | 0.9432 | . | 0.1462 | 0.2952 | 0.134 | 0.1541 | 0.3934 | 0.7894 | 0.3827 | 0.8156 | 0.2772 | 0.46 | 0.0479 | 0.1163 | 0.3651 | <.0001 | 0.4665 |
| AST | 0.5173 | 0.7796 | 0.0707 | 0.2143 | 0.2845 | 0.7957 | 0.5115 | 0.4985 | 0.1462 | . | 0.0002 | <.0001 | 0.7863 | 0.2297 | 0.5863 | 0.1315 | 0.7175 | 0.0841 | 0.696 | 0.1243 | 0.1862 | 0.1611 | 0.1257 | 0.9141 |
| ALT | 0.2394 | 0.5159 | 0.0281 | 0.3318 | 0.2294 | 0.5617 | 0.1693 | 0.2124 | 0.2952 | 0.0002 | . | 0.4318 | 0.7235 | 0.5598 | 0.9058 | 0.528 | 0.6477 | 0.4484 | 0.3373 | 0.2322 | 0.2267 | 0.4057 | 0.3124 | 0.9389 |
| γ-GTP | 0.8045 | 0.9674 | 0.9975 | 0.9278 | 0.7522 | 0.7399 | 0.6638 | 0.4985 | 0.134 | <.0001 | 0.4318 | . | 0.0539 | 0.0603 | 0.8115 | 0.4135 | 0.6948 | 0.2589 | 0.1086 | 0.8161 | 0.8431 | 0.0783 | 0.1572 | 0.866 |
| ALP | 0.1678 | 0.8462 | 0.477 | 0.5537 | 0.973 | 0.9157 | 0.7441 | 0.632 | 0.1541 | 0.7863 | 0.7235 | 0.0539 | . | 0.4398 | 0.9519 | 0.8942 | 0.9804 | 0.9681 | 0.0248 | 0.5494 | 0.584 | 0.767 | 0.2764 | 0.531 |
| LDH | 0.7922 | 0.1423 | 0.4827 | 0.3399 | 0.6693 | 0.6039 | 0.1847 | 0.1427 | 0.3934 | 0.2297 | 0.5598 | 0.0603 | 0.4398 | . | 0.2775 | 0.3589 | 0.6302 | 0.0465 | 0.0509 | 0.4074 | 0.4479 | 0.8342 | 0.1428 | 0.1448 |
| Total choresterol | 0.4756 | 0.511 | 0.5841 | 0.5982 | 0.6984 | 0.6876 | 0.0927 | 0.0629 | 0.7894 | 0.5863 | 0.9058 | 0.8115 | 0.9519 | 0.2775 | . | <.0001 | <.0001 | 0.0002 | 0.4946 | 0.492 | 0.4559 | 0.4847 | 0.2807 | 0.9686 |
| HDL | 0.4641 | 0.6665 | 0.3466 | 0.9933 | 0.1693 | 0.6761 | 0.2075 | 0.1553 | 0.3827 | 0.1315 | 0.528 | 0.4135 | 0.8942 | 0.3589 | <.0001 | . | <.0001 | 0.0001 | 0.3466 | 0.9235 | 0.6827 | 0.8272 | 0.2045 | 0.9382 |
| LDL | 0.3399 | 0.581 | 0.2584 | 0.7858 | 0.4895 | 0.832 | 0.1039 | 0.0788 | 0.8156 | 0.7175 | 0.6477 | 0.6948 | 0.9804 | 0.6302 | <.0001 | <.0001 | . | 0.0021 | 0.8023 | 0.8945 | 0.6067 | 0.4873 | 0.337 | 0.9633 |
| Triglyceride | 0.7143 | 0.6757 | 0.7314 | 0.5485 | 0.908 | 0.4151 | 0.8614 | 0.6654 | 0.2772 | 0.0841 | 0.4484 | 0.2589 | 0.9681 | 0.0465 | 0.0002 | 0.0001 | 0.0021 | . | 0.8097 | 0.468 | 0.4857 | 0.7493 | 0.2811 | 0.5101 |
| Ureiic acid | 0.9879 | 0.0626 | 0.9297 | 0.252 | 0.2202 | 0.6808 | 0.6823 | 0.7086 | 0.46 | 0.696 | 0.3373 | 0.1086 | 0.0248 | 0.0509 | 0.4946 | 0.3466 | 0.8023 | 0.8097 | . | 0.2716 | 0.1128 | 0.6272 | 0.1019 | 0.1228 |
| Na | 0.5524 | 0.289 | 0.194 | 0.0988 | 0.5426 | 0.8388 | 0.3569 | 0.3295 | 0.0479 | 0.1243 | 0.2322 | 0.8161 | 0.5494 | 0.4074 | 0.492 | 0.9235 | 0.8945 | 0.468 | 0.2716 | . | <.0001 | 0.2917 | 0.9683 | 0.9875 |
| Cl | 0.969 | 0.0294 | 0.0938 | 0.1689 | 0.3675 | 0.6749 | 0.8041 | 0.9954 | 0.1163 | 0.1862 | 0.2267 | 0.8431 | 0.584 | 0.4479 | 0.4559 | 0.6827 | 0.6067 | 0.4857 | 0.1128 | <.0001 | . | 0.6636 | 0.6438 | 0.436 |
| K | 0.395 | 0.4748 | 0.45 | 0.9171 | 0.8909 | 0.954 | 0.0031 | 0.001 | 0.3651 | 0.1611 | 0.4057 | 0.0783 | 0.767 | 0.8342 | 0.4847 | 0.8272 | 0.4873 | 0.7493 | 0.6272 | 0.2917 | 0.6636 | . | 0.5533 | 0.1266 |
| Ca | 0.1343 | 0.7036 | 0.1277 | 0.1378 | 0.0838 | 0.9372 | 0.2417 | 0.2408 | <.0001 | 0.1257 | 0.3124 | 0.1572 | 0.2764 | 0.1428 | 0.2807 | 0.2045 | 0.337 | 0.2811 | 0.1019 | 0.9683 | 0.6438 | 0.5533 | . | 0.7237 |
| serum iron | 0.7514 | 0.6481 | 0.7021 | 0.0835 | 0.4178 | 0.7213 | 0.1593 | 0.0987 | 0.4665 | 0.9141 | 0.9389 | 0.866 | 0.531 | 0.1448 | 0.9686 | 0.9382 | 0.9633 | 0.5101 | 0.1228 | 0.9875 | 0.436 | 0.1266 | 0.7237 | . |
